# Additional effect of azithromycin over β-lactam alone for severe community-acquired pneumonia-associated acute respiratory distress syndrome: a retrospective cohort study

**DOI:** 10.1186/s41479-021-00093-8

**Published:** 2022-01-10

**Authors:** Jun Suzuki, Yusuke Sasabuchi, Shuji Hatakeyama, Hiroki Matsui, Teppei Sasahara, Yuji Morisawa, Toshiyuki Yamada, Kiyohide Fushimi, Hideo Yasunaga

**Affiliations:** 1 Division of Infectious Diseases, Jichi Medical University Hospital, 3311-1 Yakushiji, Shimotsuke, Tochigi 329-0498 Japan; 2 Center for Data Science, Jichi Medical University, 3311-1 Yakushiji, Shimotsuke, Tochigi 329-0498 Japan; 3 Division of General Medicine, Jichi Medical University, 3311-1 Yakushiji, Shimotsuke, Tochigi 329-0498 Japan; 4 Department of Clinical Epidemiology and Health Economics, School of Public Health, The University of Tokyo, 7-3-1 Hongo, Bunkyo-ku, Tokyo, 113-0033 Japan; 5 Department of Infection and Immunity, School of Medicine, Jichi Medical University, 3311-1 Yakushiji, Shimotsuke, Tochigi 329-0498 Japan; 6 Department of Clinical Laboratory Medicine, Jichi Medical University, 3311-1 Yakushiji, Shimotsuke, Tochigi 329-0498 Japan; 7 Department of Health Policy and Informatics, Tokyo Medical and Dental University Graduate School of Medicine, 1-5-45, Yushima, Bunkyo-ku, Tokyo 113-8510 Japan

**Keywords:** Severe pneumonia, Azithromycin, Mortality, ARDS

## Abstract

**Background:**

Community-acquired pneumonia (CAP) is the most common cause of acute respiratory distress syndrome (ARDS). Although previous studies have suggested that macrolide therapy is beneficial for ARDS, its benefit for severe CAP-associated ARDS remains uncertain. Previous studies were limited in that they had a small sample size and included patients with non-pulmonary ARDS and those with pulmonary ARDS. This study aimed to investigate the additional effect of azithromycin when used with β-lactam compared with the effect of β-lactam alone in mechanically ventilated patients with CAP-associated ARDS.

**Methods:**

We identified mechanically ventilated patients with CAP-associated ARDS between July 2010 and March 2015 using data in the Diagnosis Procedure Combination database, a Japanese nationwide inpatient database. We performed propensity score matching analysis to assess 28-day mortality and in-hospital mortality in mechanically ventilated patients with CAP-associated ARDS who received β-lactam with and without azithromycin within hospital 2 days after admission. The inverse probability of treatment weighting analysis was also conducted.

**Results:**

Eligible patients (*n* = 1257) were divided into the azithromycin group (*n* = 226) and the control group (*n* = 1031). The one-to-four propensity score matching analysis included 139 azithromycin users and 556 non-users. No significant difference was observed between the groups with respect to 28-day mortality (34.5% vs. 37.6%, *p* = 0.556) or in-hospital mortality (46.0% vs. 49.1%, *p* = 0.569). The inverse probability of treatment weighting analysis showed similar results.

**Conclusions:**

Compared with treatment with β-lactam alone, treatment with azithromycin plus β-lactam had no significant additional effect on 28-day mortality or in-hospital mortality in mechanically ventilated patients with CAP-associated ARDS. To the best of our knowledge, this study is the first to determine the effect of azithromycin in mechanically ventilated patients with CAP-associated ARDS.

**Supplementary Information:**

The online version contains supplementary material available at 10.1186/s41479-021-00093-8.

## Introduction

Severe community-acquired pneumonia (CAP) is the most common cause of ARDS [1, 2] and death [3, 4] in critically ill patients. Approximately 80% of patients with ARDS in intensive care unit required mechanical ventilation [5] and estimates of the mortality rate in cases of severe CAP-associated ARDS have been as high as approximately 50% [2]. Strategies for treating ARDS have been investigated in many countries for reducing mortality among patients with severe CAP-associated ARDS [5].

Macrolides are a class of antibiotics that are widely used for treating CAP, and they play an important role in treating atypical pneumonia [6, 7]. Clinical and experimental studies have shown immunomodulatory effects of macrolides, such as reduction in cytokine production, neutrophil accumulation in the airways, mucus hypersecretion, and biofilm formation, as well as the acceleration of neutrophil apoptosis [8]. These effects may reduce the risk of mortality in patients with severe pneumonia [9], and several guidelines have recommended azithromycin combined with β-lactam for patients with severe CAP [6, 7].

Only few studies have investigated the effects of macrolides on severe pneumonia-associated ARDS. Some previous studies have demonstrated that macrolides, including azithromycin therapy, may be beneficial for ARDS [10–12]. However, these studies were based on small sample size and included both patients with non-pulmonary ARDS and those with pulmonary ARDS. Therefore, it remains unknown whether azithromycin has beneficial effects in patients with CAP-associated ARDS.

This study aimed to investigate the additional effect of azithromycin when used with β-lactam compared with the effect with β-lactam alone in mechanically ventilated patients with CAP-associated ARDS, using data in the Diagnosis Procedure Combination (DPC) database, a national inpatient database in Japan.

## Materials and methods

### Database information

The DPC database includes administrative claims data for approximately 8 million inpatients discharged per year from more than 1000 acute care hospitals in Japan. The attending physicians are required to accurately record the diagnoses because these diagnoses are linked to a health insurance payment system. The DPC database contains patient information on the following variables: demographics and selected clinical information: admission and discharge, discharge status (deceased or living), diagnoses, surgeries and procedures performed, medications administered, and special reimbursements for specific conditions. The DPC database also includes dates of procedures and the dosages and dates of all drugs administered during hospitalization [13, 14].

### Patient selection

**Fig. 1:**
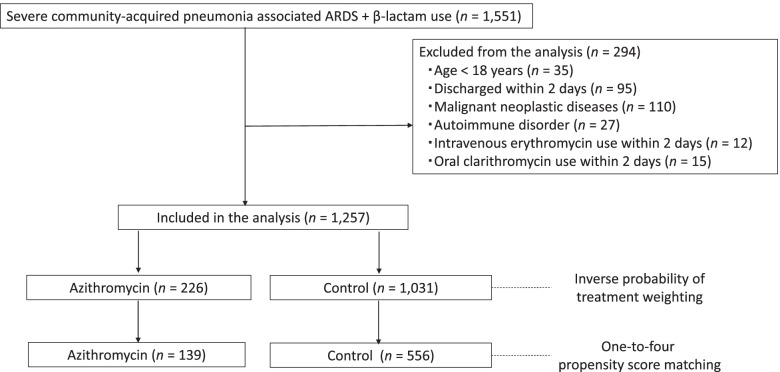
Flowchart of patient selection

In this retrospective cohort study, we identified severe CAP-associated ARDS, which was defined as mechanically ventilated patients who were diagnosed with sepsis and pneumonia and received β-lactam within hospital 2 days after admission between July 2010 and March 2015. Defining sepsis was based on the previous criteria (see Additional data 1) [14, 15]. Defining pneumonia was based on ICD-10 codes J13–J18, which is listed as the primary diagnosis or as comorbidities at admission. The patients required mechanical ventilation within hospital 2 days after admission. ARDS was defined as ICD-10 code J80 as the primary diagnosis or comorbidity at admission (Fig. 1).

The patients who meet the following criteria were excluded: < 18 years of age; discharge within hospital 2 days after admission; malignant neoplastic diseases; autoimmune disorders; intravenous erythromycin use within hospital 2 days after admission; and oral clarithromycin use within hospital 2 days after admission.

### Variables

The azithromycin group included only patients who received intravenous or oral azithromycin within hospital 2 days after admission, whereas the control group included only patients who did not receive azithromycin within hospital 2 days after admission.

Other variables that were assessed include age, sex, hospital type, hospital volume, comorbidities at admission, the Japan Coma Scale score, the age, dehydration, respiration, orientation, and blood pressure (A-DROP) system, and need for the procedures within hospital 2 days after admission. Hospital type was categorized as academic or nonacademic. Hospital volume was defined as the average annual number of mechanically ventilated patients with CAP within hospital 2 days after admission, in each hospital. The Japan Coma Scale score was recorded for all patients at admission; the level of consciousness was assessed on admission, which correlated well with the Glasgow Coma Scale [16]. We categorized the Japan Coma Scale score into four groups: 0 (alert), 1–3 (delirium), 10–30 (somnolence), and 100–300 (coma). We used the A-DROP system to assess the severity of CAP on admission. This scoring system is similar to the CURB-65 system of the British Thoracic Society and has been validated in the DPC database [17]. A-DROP severity scores were categorized into four groups: 0 (mild group), 1–2 (moderate group), 3 (severe group) and 4–5 (extremely severe group). Mild group was defined as patients who can be treated as outpatients. Moderate group was defined as patients who may be admitted to the hospital and treated. Severe group was defined as patients who should be admitted to the hospital and treated. Extremely severe group was defined as patients who require intensive care managements. Patients with missing data on A-DROP were categorized as missing on this variable. Need for the following procedures within hospital 2 days after admission was also examined: intermittent renal replacement therapy; continuous renal replacement therapy; extracorporeal membrane oxygenation; red blood cells transfusion; platelet concentrates transfusion; fresh-frozen plasma transfusion; intravenous noradrenalin; intravenous dopamine; intravenous dexamethasone; intravenous hydrocortisone; intravenous methyl prednisolone; intravenous prednisolone; intravenous antithrombin; intravenous recombinant human soluble thrombomodulin; intravenous immunoglobulin; intravenous sivelestat sodium; primary intravenous antibiotic used, divided into Ranks 1 to 5, drawing on a previous study [18] (see Additional data 2); intravenous anti-methicillin-resistant *Staphylococcus aureus* (MRSA) drugs; and intravenous fluoroquinolone.

### Outcome measures

The outcomes of this study were 28-day mortality and in-hospital mortality.

### Statistical analysis

Descriptive data are reported as numbers and percentages for categorical variables and as means and standard deviations for continuous variables. Descriptive statistics were assessed before and after propensity score matching and after inverse probability of treatment weighting (IPTW). We performed one-to-four propensity score matching to adjust for differences in baseline characteristics and disease severity on admission between the two groups. The probability that a patient received azithromycin was adjusted for potential confounders using the following characteristics: age, sex, hospital type, hospital volume, Japan Coma Scale scores, A-DROP, comorbidities at admission, renal replacement therapy, extracorporeal membrane oxygenation, transfusion, vasopressors, intravenous steroids, intravenous immunoglobulin, intravenous antithrombin, intravenous recombinant human soluble thrombomodulin, intravenous sivelestat sodium, and Rank 1-5 antibiotics, fluoroquinolone, and anti-MRSA drugs used. The following interaction terms were added to estimate the propensity score to achieve a better balance in patient characteristics between the two groups: age and mild A-DROP score, age and dopamine, age and somnolence as consciousness level and extracorporeal membrane oxygenation, and fluoroquinolone and anti-MRSA drugs. Several elements from the patients’ medical histories (severe liver disease, hemiplegia, or paraplegia) and initial use of several drugs (clindamycin, aminoglycoside, tetracycline, and anti-fungal drugs within hospital 2 days after admission,) were not included in this analysis because they were relevant only to a few patients. We used absolute standardized mean differences and assessed the balance in patient characteristics [19]. Absolute standardized mean differences of less than 0.1 were considered negligible imbalances in baseline characteristics and disease severity on admission between the groups [20]. Fisher’s exact test was performed to compare 28-day mortality and in-hospital mortality between the groups. We used IPTW to estimate the treatment effect [21]. We calculated risk differences and their 95% CIs between the before and after propensity score-matched, and after IPTW analyses [21]. The level of statistical significance was *P* <  0.05 for a two-sided test. Propensity score matching was conducted with the “matching” package, and IPTW analysis was conducted with the “survey” package in R statistical software, Version 3.1.3 (The R Foundation, Vienna, Austria). All other analyses were conducted using IBM SPSS, Version 25 (IBM SPSS, Armonk, NY).

## Results

A total of 1257 patients with severe CAP-associated ARDS during the 57-month study period were identified in the DPC database. The eligible patients were divided into the azithromycin group (*n* = 226) and the control group (*n* = 1031). The one-to-four propensity score matching analysis included 139 azithromycin users (azithromycin group) and 556 non-users (control group).

### Baseline characteristics

Table 1 reveals the baseline characteristics of the before and after propensity score-matched groups. After propensity score matching, there was adequate balance in patient characteristics between the groups.

**Table 1:** Patients’ baseline characteristics in before and after propensity score-matched groups

	Before propensity score-matched groups			After propensity score-matched groups		
	Azithromycin group	Control group	SMD	Azithromycin group	Control group	SMD
Variable	*n* = 226	*n* = 1031		*n* = 139	*n* = 556	
Age (years), mean (SD)	70.0 (14.8)	73.6 (12.9)	0.25	71.8 (13.7)	72.7 (12.9)	0.07
Sex (female), *n* (%)	64 (28.3)	316 (30.6)	0.05	39 (28.1)	161 (29.0)	0.02
Hospital type (academic), *n* (%)	50 (22.1)	162 (15.7)	0.16	30 (21.6)	99 (17.8)	0.09
Hospital volume (cases/year), mean (SD)	118.0 (58.6)	106.9 (60.8)	0.19	108.9 (48.9)	111.1 (64.7)	0.04
**Comorbidities**, *n* (%**)**						
Myocardial infarction	3 (1.3)	15 (1.5)	0.01	2 (1.4)	3 (0.5)	0.09
Congestive heart failure	45 (19.9)	239 (23.2)	0.08	33 (23.7)	122 (21.9)	0.04
Peripheral vascular disease	7 (3.1)	11 (1.1)	0.14	3 (2.2)	9 (1.6)	0.04
Cerebrovascular disease	9 (4.0)	69 (6.7)	0.12	8 (5.8)	34 (6.1)	0.02
Dementia	1 (0.4)	25 (2.4)	0.17	1 (0.7)	3 (0.5)	0.02
Chronic pulmonary disease	18 (8.0)	93 (9.0)	0.04	11 (7.9)	51 (9.2)	0.05
Peptic ulcer	6 (2.7)	34 (3.3)	0.04	4 (2.9)	15 (2.7)	0.01
Mild liver disease	7 (3.1)	29 (2.8)	0.02	4 (2.9)	17 (3.1)	0.01
Diabetes without chronic complications	30 (13.3)	164 (15.9)	0.08	21 (15.1)	82 (14.7)	0.01
Diabetes with chronic complications	12 (5.3)	48 (4.7)	0.03	8 (5.8)	26 (4.7)	0.05
Renal disease	12 (5.3)	74 (7.2)	0.08	10 (7.2)	37 (6.7)	0.02
**Consciousness level**, *n* (%)						
Alert	136 (60.2)	517 (50.1)	0.20	79 (56.8)	307 (55.2)	0.03
Delirium	48 (21.2)	232 (22.5)	0.03	32 (23.0)	120 (21.6)	0.04
Somnolence	11 (4.9)	100 (9.7)	0.19	9 (6.5)	34 (6.1)	0.02
Coma	26 (11.5)	149 (14.5)	0.09	17 (12.2)	78 (14.0)	0.05
**A-DROP category**, *n* (%)						
Mild	1 (0.3)	5 (0.5)	0.01	0 (0.0)	2 (0.4)	0.09
Moderate	28 (12.4)	83 (8.1)	0.14	14 (10.1)	54 (9.7)	0.01
Severe	19 (8.4)	86 (8.3)	< 0.01	12 (8.6)	50 (9.0)	0.01
Extremely severe	25 (11.1)	185 (17.9)	0.20	17 (12.2)	84 (15.1)	0.08
Missing	154 (68.1)	677 (65.7)	0.05	96 (69.1)	368 (66.2)	0.06
**Intervention**, *n* (%)						
Renal replacement therapy	36 (15.9)	102 (9.9)	0.18	15 (10.8)	59 (10.6)	0.01
Extracorporeal membrane oxygenation	14 (6.2)	9 (0.9)	0.29	1 (0.7)	3 (0.5)	0.02
**Catecholamines**, *n* (%)						
Dopamine	55 (24.3)	373 (36.2)	0.26	37 (26.6)	169 (30.4)	0.08
Noradrenaline	101 (44.7)	344 (33.4)	0.23	52 (37.4)	212 (38.1)	0.02
**Transfusion,** ***n*** **(%)**						
Red cell transfusion	18 (8.0)	97 (9.4)	0.05	12 (8.6)	40 (7.2)	0.05
Platelets transfusion	7 (3.1)	34 (3.3)	0.01	1 (0.7)	10 (1.8)	0.09
Fresh frozen plasma transfusion	4 (1.8)	41 (4.0)	0.13	2 (1.4)	8 (1.4)	< 0.01
**Other treatment,** ***n*** **(%)**						
Antithrombin	30 (13.3)	92 (8.9)	0.14	12 (8.6)	47 (8.5)	0.01
Recombinant human soluble thrombomodulin	36 (15.9)	92 (8.9)	0.21	11 (7.9)	58 (10.4)	0.09
Immunoglobulin	54 (23.9)	238 (23.1)	0.02	36 (25.9)	126 (22.7)	0.08
Sivelestat sodium	101 (44.7)	532 (51.6)	0.14	68 (48.9)	259 (46.6)	0.05
Steroid	108 (47.8)	458 (44.4)	0.07	60 (43.2)	251 (45.1)	0.04
**Initial antibiotic**, *n* (%)						
Rank 5	110 (48.7)	586 (56.8)	0.16	73 (52.5)	286 (51.4)	0.02
Rank 4	63 (27.9)	246 (23.9)	0.09	41 (29.5)	149 (26.8)	0.06
Rank 3	63 (27.9)	164 (15.9)	0.29	27 (19.4)	119 (21.4)	0.05
Rank 2	27 (11.9)	153 (14.8)	0.09	18 (12.9)	76 (13.7)	0.02
Rank 1	7 (3.1)	31 (3.0)	0.01	3 (2.2)	12 (2.2)	< 0.01
Anti-MRSA drug	18 (8.0)	79 (7.7)	0.01	12 (8.6)	36 (6.5)	0.08
Fluoroquinolone	37 (16.4)	376 (36.5)	0.47	34 (24.5)	121 (21.8)	0.06

*Abbreviations*: *A-DROP* severity score consisting of age, dehydration, respiration, orientation, and blood pressure, *MRSA* methicillin-resistant *Staphylococcus aureus*, *SD* standard deviation, *SMD* standardized mean difference

### Outcome measures

Table 2 reveals the results of 28-day mortality and in-hospital mortality in the azithromycin and control groups before and after propensity score matching. Before propensity score matching, the 28-day mortality rate was 36.3% in the azithromycin group vs. 36.9% in the control group (*p* = 0.939), and the in-hospital mortality rate was 46.0% in the azithromycin group vs. 49.7% in the control group (*p* = 0.340). After propensity score matching, there were no significant differences between the two groups for 28-day mortality (34.5% vs. 37.6%, *p* = 0.556) or in-hospital mortality (46.0% vs. 49.1%, *p* = 0.569). The IPTW analysis also revealed similar results (see Additional data 3 and Additional data 4).

**Table 2:** Twenty-eight-day mortality and in-hospital mortality in before and after propensity score-matched groups

	Before propensity-score matched group			After propensity-score matched group		
	Azithromycin group	Control group	P	Azithromycin group	Control group	P
Outcome, n	226	1031		139	556	
Twenty-eight-day mortality, *n* (%)	82 (36.3)	380 (36.9)	0.939	48 (34.5)	209 (37.6)	0.556
In-hospital mortality, *n* (%)	104 (46.0)	512 (49.7)	0.340	64 (46.0)	273 (49.1)	0.569

## Discussion

The present study using a DPC database showed that the addition of azithromycin to β-lactam treatment was not associated with a lower rate of 28-day mortality or in-hospital mortality, compared with the rate in treatment with β-lactam alone, in mechanically ventilated patients with CAP-associated ARDS.

Several possible explanations for these results should be considered. First, the immunomodulatory effects of azithromycin may not reduce inflammatory markers associated with ARDS or mortality among mechanically ventilated patients with CAP-associated ARDS. A previous experimental study evaluated the immunomodulatory effects of macrolides using total cell count and neutrophil count in bronchoalveolar lavage fluids in mice with bleomycin-induced acute lung injury. The results showed that, compared with other macrolide drugs, azithromycin was less active in inhibiting neutrophil cells, which caused acute lung injury [22]. This may also explain our results.

Second, atypical pathogens such as *Mycoplasma pneumoniae* or *Chlamydophila pneumoniae* as target organisms of azithromycin rarely cause progression to ARDS, which may have influenced our results. Previous studies have shown that a few patients with atypical pneumonia showed progression to severe CAP or ARDS [23] and that approximately 5% of atypical organisms cause CAP-associated ARDS [24, 25]. Thus, there may have been few patients in our sample for whom β-lactam was insufficient and for whom the addition of azithromycin was effective, and this may be the reason why azithromycin did not reduce mortality in patients with CAP-associated ARDS in our study.

Third, the difference in the susceptibility of pneumonia pathogens may also explain our results. The addition of azithromycin to β-lactam has been reported to be double coverage of *Streptococcus pneumoniae*, which is the leading pathogen of CAP-associated ARDS [26]. However, approximately 80% of *S. pneumoniae* strains are resistant to azithromycin in Japan [2, 27]. Several guidelines recommended macrolide use for pneumonia for the following reasons except double coverage of *S. pneumoniae*: treating atypical pneumonia pathogens, or immunomodulatory effects [6, 7, 26]. In a population where rate of atypical pathogens, etiology, and azithromycin resistance levels differ, the value of azithromycin therapy could be higher or lower. Therefore, in Japan, the value of azithromycin therapy might be lower and avoiding the use of azithromycin as immunomodulatory effects for ARDS might also help reduce azithromycin resistance levels.

Several strengths exist in this study. First, the results of our study was conducted in a real-world clinical setting in Japan. Second, our study included approximately 50% of inpatients who were admitted to acute care hospitals in Japan during the 57-month study period. Third, this is the first study to research the effect of azithromycin in mechanically ventilated patients with CAP-associated ARDS to the best of our knowledge.

Several limitations also exist in this study. First, the DPC database lacks detailed clinical data such as information on vital signs, results of blood tests, which may have biased the results. Second, the DPC database lacks of information on blood or respiratory cultures of the organisms that caused CAP-associated ARDS and the lack of the information may have influenced the results. Third, the antibiotic susceptibilities of organisms that caused CAP-associated ARDS may differ between Japan and other countries. Therefore, our results might not be generalizable to other countries. Fourth, the DPC database lacks data on the mechanical ventilation setting. Therefore, we did not have information on the severity of ARDS based on the Berlin criteria, which may have biased the results. Fifth, our study included ventilated patients with ARDS; however, we did not include ARDS patients, and the results may be different between ventilator or non-ventilator required ARDS. Sixth, the DPC database lacks detailed clinical data such as pneumococcal vaccine and rate of pneumococcal vaccination in Japan may differ from abroad, which may be biased the results. Finally, we conducted propensity score matching analysis and IPTW to adjust for patient background characteristics and there was adequate balance in patient characteristics between the azithromycin group and the control group in this study. One of the assumption using propensity score matching analysis is the strongly ignorable treatment assignment. If there are the strongly ignorable treatment assignment and no unmeasured confounders, conditioning on the propensity score can result in unbiased estimates of average treatment effects. However, this assumption is untestable, and the confounding due to unmeasured covariates cannot be avoided completely. Therefore, the confounding due to unmeasured covariates may have biased the results.

## Conclusions

This Japanese nationwide inpatient database showed that the addition of azithromycin to β-lactam treatment was not associated with a lower rate of 28-day mortality or in-hospital mortality compared with the rate in treatment with β-lactam alone in mechanically ventilated patients with CAP-associated ARDS. There is not a strong enough rationale to recommend or not to recommend the use of azithromycin for ARDS to clinical guidelines, and further clinical studies are needed to confirm this result.

## Supplementary Information

**Additional file 1: Data 1**. ICD-10 codes for sepsis.

**Additional file 2: Data 2**. Classification of β-lactam antibiotics.

**Additional file 3: Table 3**. Patients’ characteristics in the IPTW analysis groups.

**Additional file 4: Table 4**. Risk differences for 28-day mortality and in-hospital mortality in the unmatched, propensity score-matched, and IPTW analysis groups.

## Data Availability

The datasets used and analyzed during the current study are not publicly available for ethical reasons as the data are patient data. The data are available to interested researchers on reasonable request to the corresponding author, pending ethical approval.

## Abbreviations

A-DROP: Age dehydration respiration orientation and blood pressure
ARDS: Acute respiratory distress syndrome
CAP: Community-acquired pneumonia
DPC: Diagnosis Procedure Combination
ICD-10: International Classification of Diseases, Tenth Revision
IPTW: Inverse probability of treatment weighting
MRSA: Methicillin-resistant *Staphylococcus aureus*
SD: Standard deviation
SMD: Standardized mean difference
