# Supplementary material for: Additional effect of azithromycin over β-lactam alone for severe community-acquired pneumonia-associated acute respiratory distress syndrome: a retrospective cohort study
Source: Pneumonia (Nathan). 2022 Jan 10;14:1. doi: 10.1186/s41479-021-00093-8 (PMC8744237; doi:10.1186/s41479-021-00093-8)
Supplement: Supplementary file 2 — Additional file 2: Data 2. Classification of β-lactam antibiotics. [file 41479_2021_93_MOESM2_ESM.docx]

**Additional data 2.** Classification of β-lactam antibiotics

| Rank 5: carbapenems | biapenem, doripenem, imipenem/cilastatin, meropenem, panipenem/betamipron |
| --- | --- |
| Rank 4: antipseudomonal β-lactams | piperacillin, piperacillin/tazobactam, cefepime, cefoperazone/sulbactam, cefozopran, ceftazidime, aztreonam |
| Rank 3: third-generation cephems | cefmenoxime, cefodizime, cefotaxime, ceftriaxone, latamoxef |
| Rank 2: ampicillin with β-lactamase inhibitor | ampicillin/sulbactam |
| Rank 1: other β-lactams | penicillin G, ampicillin, cefalotin, cefazolin, cefmetazole, cefminox, cefotiam, flomoxef |
