# Supplementary material for: Additional effect of azithromycin over β-lactam alone for severe community-acquired pneumonia-associated acute respiratory distress syndrome: a retrospective cohort study
Source: Pneumonia (Nathan). 2022 Jan 10;14:1. doi: 10.1186/s41479-021-00093-8 (PMC8744237; doi:10.1186/s41479-021-00093-8)
Supplement: Supplementary file 3 — Additional file 3: Table 3. Patients’ characteristics in the IPTW analysis groups. [file 41479_2021_93_MOESM3_ESM.docx]

**Additional data 3.** Patients’ characteristics in the IPTW analysis groups

|  | IPTW analysis groups | |  |
| --- | --- | --- | --- |
|  | Azithromycin  group | Control  group | SMD |
| Variable | n = 1238.5 | n = 1255.8 |  |
| Age (years), mean (SD) | 73.0 (12.8) | 72.9 (13.4) | 0.01 |
| Sex (female), *n* (%) | 373.5 (30.2) | 379.6 (30.2) | < 0.01 |
| Hospital type (academic), *n* (%) | 234.1 (18.9) | 212.5 (16.9) | 0.05 |
| Hospital volume (cases/year), mean (SD) | 111.5 (55.1) | 109.0 (61.9) | 0.04 |
| **Comorbidities**, *n* (%**)** |  |  |  |
| Myocardial infarction | 8.2 (0.7) | 17.2 (1.4) | 0.07 |
| Congestive heart failure | 233.7 (18.9) | 284.2 (22.6) | 0.09 |
| Peripheral vascular disease | 16.4 (1.3) | 21.8 (1.7) | 0.03 |
| Cerebrovascular disease | 84.7 (6.8) | 78.4 (6.2) | 0.02 |
| Dementia | 11.8 (1.0) | 26.0 (2.1) | 0.09 |
| Chronic pulmonary disease | 113.5 (9.2) | 110.0 (8.8) | 0.01 |
| Peptic ulcer | 42.5 (3.4) | 40.0 (3.2) | 0.01 |
| Mild liver disease | 30.8 (2.5) | 35.3 (2.8) | 0.02 |
| Diabetes without chronic complications | 225.3 (18.2) | 193.0 (15.4) | 0.08 |
| Diabetes with chronic complications | 42.8 (3.5) | 57.4 (4.6) | 0.06 |
| Renal disease | 78.9 (6.4) | 86.3 (6.9) | 0.02 |
| **Consciousness level**, *n* (%) |  |  |  |
| Alert | 647.7 (52.3) | 652.6 (52.0) | < 0.01 |
| Delirium | 290.3 (23.4) | 280.7 (22.4) | 0.03 |
| Somnolence | 77.8 (6.3) | 110.7 (8.8) | 0.09 |
| Coma | 203.5 (16.4) | 174.9 (13.9) | 0.07 |
| **A-DROP category**, *n* (%) |  |  |  |
| Mild | 2.4 (0.2) | 5.8 (0.5) | 0.05 |
| Moderate | 107.3 (8.7) | 113.4 (9.0) | 0.01 |
| Severe | 108.7 (8.8) | 106.0 (8.4) | 0.01 |
| Extremely severe | 193.3 (15.6) | 208.5 (16.6) | 0.03 |
| Missing | 829.3 (67.0) | 827.9 (65.9) | 0.02 |
| **Intervention**, *n* (%) |  |  |  |
| Renal replacement therapy | 107.8 (8.7) | 132.3 (10.5) | 0.06 |
| Extracorporeal membrane oxygenation | 19.8 (1.6) | 18.6 (1.5) | 0.01 |
| **Catecholamines**, *n* (%) |  |  |  |
| Dopamine | 428.0 (34.6) | 426.9 (34.0) | 0.01 |
| Noradrenaline | 470.6 (38.0) | 445.0 (35.4) | 0.05 |
| **Transfusion, *n* (%)** |  |  |  |
| Red cell transfusion | 109.8 (8.9) | 119.2 (9.5) | 0.02 |
| Platelets transfusion | 40.6 (3.3) | 41.3 (3.3) | < 0.01 |
| Fresh frozen plasma transfusion | 48.5 (3.9) | 45.3 (3.6) | 0.02 |
| **Other treatment, *n* (%)** |  |  |  |
| Antithrombin | 120.7 (9.7) | 124.0 (9.9) | < 0.01 |
| Recombinant human soluble thrombomodulin | 157.9 (12.7) | 130.9 (10.4) | 0.07 |
| Immunoglobulin | 325.3 (26.3) | 292.0 (23.3) | 0.07 |
| Sivelestat sodium | 668.5 (54.0) | 637.9 (50.8) | 0.06 |
| Steroid | 520.3 (42.0) | 568.2 (45.2) | 0.07 |
| **Initial antibiotic**, *n* (%) |  |  |  |
| Rank 5 | 657.4 (53.1) | 694.5 (55.3) | 0.05 |
| Rank 4 | 300.8 (24.3) | 311.1 (24.8) | 0.01 |
| Rank 3 | 253.5 (20.5) | 225.8 (18.0) | 0.06 |
| Rank 2 | 150.7 (12.2) | 177.3 (14.1) | 0.06 |
| Rank 1 | 26.1 (2.1) | 35.3 (2.8) | 0.05 |
| Anti-MRSA drug | 90.2 (7.3) | 97.2 (7.7) | 0.02 |
| Fluoroquinolone | 426.2 (34.4) | 416.7 (33.2) | 0.03 |

*Abbreviations*: A-DROP: severity score consisting of age, dehydration, respiration, orientation, and blood pressure; IPTW: inverse probability of treatment weighting; MRSA: methicillin-resistant *Staphylococcus aureus*; SD: standard deviation; SMD: standardized mean difference.
