# Supplementary material for: Additional effect of azithromycin over β-lactam alone for severe community-acquired pneumonia-associated acute respiratory distress syndrome: a retrospective cohort study
Source: Pneumonia (Nathan). 2022 Jan 10;14:1. doi: 10.1186/s41479-021-00093-8 (PMC8744237; doi:10.1186/s41479-021-00093-8)
Supplement: Supplementary file 4 — Additional file 4: Table 4. Risk differences for 28-day mortality and in-hospital mortality in the unmatched, propensity score-matched, and IPTW analysis groups. [file 41479_2021_93_MOESM4_ESM.docx]

**Additional data 4.** Risk differences for 28-day mortality and in-hospital mortality in the before and after propensity score-matched, and IPTW analysis groups

|  | Before propensity-score matched group | P | After propensity-score matched group | P | IPTW analysis group | P |
| --- | --- | --- | --- | --- | --- | --- |
| 28-day mortality | -0.6%  (-7.5% to 6.3%) | 0.939 | -3.1%  (-11.9% to 5.8%) | 0.556 | -0.3%  (-9.6% to 9.0%) | 0.949 |
| In-hospital  mortality | -3.6%  (-10.8% to 3.5%) | 0.340 | -3.1%  (-12.3% to 6.2%) | 0.569 | -1.1%  (-10.9% to 8.6%) | 0.817 |

*Abbreviation*: IPTW: inverse probability of treatment weighting
